# Machine learning methods for propensity and disease risk score estimation in high-dimensional data: a plasmode simulation and real-world data cohort analysis

**DOI:** 10.3389/fphar.2024.1395707

**Published:** 2024-10-28

**Authors:** Yuchen Guo, Victoria Y. Strauss, Martí Català, Annika M. Jödicke, Sara Khalid, Daniel Prieto-Alhambra

**Affiliations:** ^1^ Pharmaco- and Device Epidemiology Group, Centre of Statistics in Medicine, Nuffield Department of Orthopaedics, Rheumatology and Musculoskeletal Sciences (NDORMS), University of Oxford, Oxford, United Kingdom; ^2^ Boehringer-Ingelheim, Ingelheim, Germany; ^3^ Department of Medical Informatics, Erasmus Medical Center, Rotterdam, Netherlands

**Keywords:** treatment effect, observational research, machine learning, propensity scores, disease risk scores, negative control

## Abstract

**Introduction:**

Machine learning (ML) methods are promising and scalable alternatives for propensity score (PS) estimation, but their comparative performance in disease risk score (DRS) estimation remains unexplored.

**Methods:**

We used real-world data comparing antihypertensive users to non-users with 69 negative control outcomes, and plasmode simulations to study the performance of ML methods in PS and DRS estimation. We conducted a cohort study using UK primary care records. Further, we conducted a plasmode simulation with synthetic treatment and outcome mimicking empirical data distributions. We compared four PS and DRS estimation methods: 1. Reference: Logistic regression including clinically chosen confounders. 2. Logistic regression with L1 regularisation (LASSO). 3. Multi-layer perceptron (MLP). 4. Extreme Gradient Boosting (XgBoost). Covariate balance, coverage of the null effect of negative control outcomes (real-world data) and bias based on the absolute difference between observed and true effects (for plasmode) were estimated. 632,201 antihypertensive users and nonusers were included.

**Results:**

ML methods outperformed the reference method for PS estimation in some scenarios, both in terms of covariate balance and coverage/bias. Specifically, XgBoost achieved the best performance. DRS-based methods performed worse than PS in all tested scenarios.

**Discussion:**

We found that ML methods could be reliable alternatives for PS estimation. ML-based DRS methods performed worse than PS ones, likely given the rarity of outcomes.

## 1 Introduction

Observational studies complement randomised controlled trials in assessing medicine and vaccine risks and benefits. Large healthcare records, known as “real-world data,” offer insights into medical interventions in diverse populations but can introduce bias (Ryan et al., 2012; Rosenbaum and Rubin, 1983). In causal inference, propensity scores (PS) and disease risk scores (DRS) are used to mitigate confounding by balancing covariates between treated and untreated groups, enabling treatment effect estimation. Both PS and DRS can be used with methods like matching and weighting to estimate causal treatment effects (Desai et al., 2015; Nguyen et al., 2024; Rosenbaum and Rubin, 1983; Lee et al., 2010; Wyss et al., 2015).

PS, estimating the probability of receiving treatment based on covariates, mitigate confounding in pharmaco-epidemiology (Austin, 2011). PS are often estimated using logistic regression with predetermined confounders based on previous clinical knowledge. Logistic regression with L1 regularisation (LASSO), a data-driven method, is increasingly popular and well implemented for PS estimation (Ryan et al., 2013; Tian et al., 2018; Greenland, 2008). While LASSO has shown good performance and scalability in PS estimation, various machine learning (ML) methods, including neural networks and tree-based methods can be applied. (Abdia et al., 2017; Cannas and Arpino, 2019; Collier et al., 2023; Lee et al., 2010; Setoguchi et al., 2008). We selected Multi-layer perceptron (MLP) and Extreme Gradient Boosting (XgBoost) for their distinct advantages. MLP is capable of modelling complex, non-linear relationships, making it suitable for capturing interactions between covariates in high-dimensional data (Setoguchi et al., 2008). XgBoost is well-known for its effectiveness in handling large data and robust performance, while boosting methods consistently highlighted in the literature for PS estimation (Chen and Guestrin, 2016; Lee et al., 2010; Abdia et al., 2017). These characteristics make both MLP and XgBoost appropriate choices for our large data, where optimising hyperparameters is crucial to avoid the pitfalls of using default settings (Collier et al., 2023).

DRS, based on the estimated probability of outcome given confounders, offer an alternative to PS for confounding mitigation. Less popular than PS, DRS have shown worse performance than PS in some simulation studies (Wyss et al., 2015; Xu et al., 2016), but are easier to understand and interpret as they represent disease severity or outcome risk. However, the potential of ML methods for DRS estimation in treatment effect estimation has not been widely investigated.

Although LASSO has been extensively studied with respect to hyperparameter tuning, most non-regularisation-based ML methods, such as neural networks and tree-based algorithms, have often been applied using default settings in PS estimation. To our best knowledge, among studies using simulation for methodology research, only Collier et al. (2023) and Weberpals et al. (2021) tuned a neural network model, Vegetabile et al. (2020) tuned a Gaussian processes model, and Sales et al. (2018) tuned a random forest model. No study has yet compared regression-based methods with well-tuned ML methods when comparing PS and DRS method. Recent study investigating into hyperparameter tuning suggest that tuning ML method produced more accurate treatment effect estimation (Amusa, North and Zewotir, 2023). This highlights the importance of our study within the field of ML applications for PS and DRS estimation.

We aimed to demonstrate the use of various ML methods for PS and DRS estimation in the context of large real-world data and plasmode simulations. We compared logistic regression informed by previous knowledge with three data-driven ML methods: LASSO, MLP, and XgBoost. The real-world data analysis explored the association between antihypertensive treatment and negative control outcomes. Plasmode simulations were conducted to mimic real-world data but with known true treatment effects.

## 2 Methods

### 2.1 Real-world data

First, we conducted a real-world data analysis of the effects of antihypertensives on fracture risk in elderly people (see Study Population Section 2.1.2). Since the true causal relationship between antihypertensives and fractures is unknown, we used negative control outcomes to evaluate potential bias in different methods. We modelled 69 negative control outcomes (See Supplementary Material for list) selected based on clinical expertise, while the clinical outcome (fracture) was only used to identify confounders for the reference method in PS and DRS estimation.

#### 2.1.1 Data source

Data was obtained from the Clinical Practice Research Datalink (CPRD GOLD) (Herrett et al., 2015), a UK primary care database with a representative sample of over 6 million people active during the study period. Data were mapped to the Observational Medical Outcomes Partnership Common Data Model (OMOP) (Stang et al., 2010).

#### 2.1.2 Study population

The source population included individuals aged over 65 at the beginning of the study on 1 January 2010. These individuals had to be registered with a medical practice meeting the “up-to-standard” criteria for at least 1 year. Those who had taken antihypertensive/s in the year preceding the study start were excluded. Additionally, a minimum follow-up of 1 day was required.

#### 2.1.3 Exposure

Antihypertensive treatment episodes were generated using Gardarsdottir et al. (2010) method by concatenating prescriptions with a *<* 90-day refill gap and used as a time-varying exposure.

Participants started as “non-users” (1/1/2010), until antihypertensive initiation. The first prescription marked the shift to “drug user” status, maintained until the end of follow-up. Details of the study design can be found in Supplementary Material.

#### 2.1.4 Outcome

First, the main outcome of clinical interest was identified using previously used codes for fractures (see Supplementary Material). This outcome was only used for expert selection of confounders for the reference logistic model, as the unknown true effect size makes method comparison infeasible, we did not include the estimation of treatment effect on outcome in the main result. Nonetheless, the estimated effect size of antihypertensive treatment on fractures is provided in the Supplementary Material for reference.

To measure bias and compare model performance in the real-world data analysis with an unknown treatment effect, we conducted a negative outcome control analysis using 69 outcomes presumed to have no causal relation with antihypertensive treatment (Lipsitch et al., 2010). These outcomes, expected to have a true hazard ratio of 1, served as a benchmark. The coverage of the expected null effect within confidence intervals for each of these negative control outcomes acted as a proxy for bias. See Supplementary Material for the list of proposed negative control outcomes.

#### 2.1.5 Covariates

To estimate PS and DRS in the real-world data analysis, we used all available information of relevance in CPRD GOLD, including demographics, year of treatment initiation, conditions, procedures, and drugs (Marc Overhage et al., 2012), after excluding covariates with low prevalence (≤0.004). Details of these can be found in Supplementary Material.

### 2.2 Plasmode simulation

Second, we conducted a plasmode simulation (Franklin et al., 2014) by generating synthetic exposures and outcomes based on the essential covariates and distributions observed in the previous real-world data. Using this method, we conducted a similar cohort analysis to investigate the effect of time-varying synthetic exposure among users versus non-users in relation to a synthetic outcome. Details are in Supplementary Material.

#### 2.2.1 Data source and study population

Plasmode simulations used in this study were based on resampling with replacement of the observed covariates and controlling the treatment effect via parameters for the outcome associated with covariates, ensuring that associations among covariates were representative of real-world scenarios. The same covariates and observations from real-world data introduced above were selectively used in plasmode simulation.

#### 2.2.2 Exposure and outcome generation

Exposure and outcome were simulated based on confounders (affecting both treatment and outcome), instrumental variables (only affecting treatment) and risk factors (only affecting outcome) selected. Details can be found in Supplementary Material.

#### 2.2.3 Covariates

90 covariates were selected from the data generated above to be covariates that affect treatment or outcome or both. Among 90 covariates, 50 of them were confounders, 20 of them were instrumental variables, and 20 of them were risk factors.

### 2.3 Machine learning and reference methods

In the plasmode simulation, as explained above, the reference method incorporated true confounders that affect both treatment and outcome, representing the clinically informed covariates. Other ML methods used for PS and DRS estimation considered all covariates as input and selected relevant covariates through a data-driven modelling approach. Specifically, we applied LASSO, MLP and XgBoost. LASSO is a form of logistic regression that includes an L1 regularisation term, which adds a constraint to the model that shrinks the coefficients of less important variables to zero (Tibshirani, 1996). This helps with variable selection and regularisation, reducing overfitting while maintaining interpretability. MLP is a type of feedforward neural network, which consists of multiple layers of interconnected neurons (Rumelhart et al., 1986; Goodfellow et al., 2016). Each neuron performs a weighted sum of its inputs followed by an activation function to introduce non-linearity. XgBoost is a tree-based ensemble method that builds decision trees sequentially, with each tree attempting to correct the errors made by the previous ones (Chen and Guestrin, 2016). It uses a gradient descent approach to minimise the loss function, making it highly effective for tasks with structured data.

Each model was hyperparameter-tuned using 10-fold cross-validation, aiming to minimise the average Brier score across the folds. For LASSO, the shrinkage parameter was optimised. MLP tuning included the optimiser, batch size, number of epochs, kernel function, number of hidden layers, number of units in each layer, and activation function. For XgBoost, we tuned parameters such as the number of estimators, minimum sum of instance weight (hessian) in a child, minimum loss reduction for partitioning, subsample ratio, learning rate, and maximum tree depth. The Python codes used for implementation and full details on hyperparameter tuning are available in the GitHub repository (https://github.com/MimimimiGuo/plasmode) and in Supplementary Material for reproducibility.

### 2.4 Propensity scores and disease risk scores settings

PS represents the probability of receiving treatment conditional on confounders. Many PS methods have been tested to reduce confounding effects (Austin, 2011; Ali et al., 2016).

DRS, proposed by Miettinen (Miettinen, 1976), addresses confounding by conditioning on the estimated probability of outcome, calculated either as an *unexposed DRS* or a *full cohort DRS*. The *unexposed DRS* is computed by regressing the outcome to covariates *Y* ∼ *X* |*T* = 0 for the unexposed population, then extending the model to the entire population, resulting in fitted values *P*(*Y* = 1 | *X*) as the unexposed DRS. The *full cohort DRS* is obtained by regressing the outcome to covariates and treatments using the entire study population *Y* ∼ *X, T*, and computing fitted values for the full population by setting treatment status to unexposed *T* = 0. Additionally, methods like out-of-sample DRS approaches have been explored to apply the DRS to external populations for improved generalisability (Wyss et al., 2014), such extensions are beyond the scope of this study. For this study, we used the *full cohort DRS* as it is known to outperform the *unexposed DRS* in reducing bias when estimating treatment effects (Arbogast and Ray, 2011).

A greedy matching method (Rassen et al., 2012) was applied with a maximum ratio of 5:1 and a caliper of 0.05, following the study done by Tian et al. (2018) on large scale PS estimation. After matching, we obtained the average treatment effect on the treated (ATT).

In the real-world data, we applied Cox regression to estimate hazard ratios for treatment effects on negative control outcomes using matched data. In the plasmode simulation data, logistic regression was applied to matched data, with treatment as the only covariate and the simulated outcome as the response variable.

### 2.5 Estimates and metrics

Average Absolute Standardised Mean Difference (ASMD) measured covariate balance across all the available covariates in the real-world data and plasmode simulation data.

The coverage and root mean square error (RMSE) of the estimated hazard ratio for negative control outcomes was reported for real-world data analysis, where a true hazard ratio of one is assumed.

Relative bias with 95% confidence intervals was used as a metric to evaluate the accuracy of treatment effect estimation in the plasmode analysis, and it was presented on the scale of the treatment variable’s coefficients. It is calculated as

$$\frac{\left|\;{\hat{\beta }}_{t}-\beta_{t}\right|}{\beta_{t}}$$

if denoting *β*_*t*_ as the true treatment effect coefficient and ${\hat{\beta }}_{t}$ as the estimated treatment effect.

In addition to these metrics, we used Brier score loss after 10-fold cross-validation as an out-of-sample performance measure for ML methods (see Supplementary Material). Together, these metrics allow for a comprehensive evaluation of model performance in estimating PS, DRS and treatment effects.

## 3 Results

### 3.1 Cohort

A total of 163,597 antihypertensive drug users and 468,604 non-users were included from CPRD data, with 637 baseline covariates available for PS/DRS estimation and outcome risk of 0.0075. Details of plasmode data generation are available in Supplementary Material.

### 3.2 Propensity scores results

#### 3.2.1 Propensity scores covariate balance in real-world data and plasmode simulation

**FIGURE 1:**
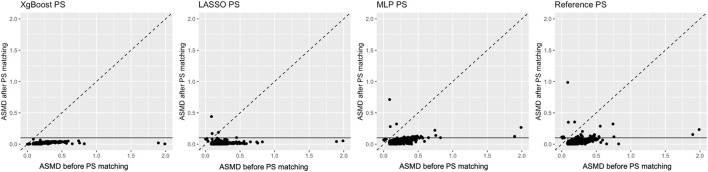
Average standardised absolute mean difference before (*X*-axis) and after propensity score matching (*Y*-axis) in real-world data. The horizontal line highlights the pre-specified threshold for the desired average standardised absolute mean difference below 0.1 after matching.

Covariate balance was evaluated in both real-world data and plasmode simulation analyses through ASMD after matching (Table 1). Details of the balance for each covariate before and after PS matching are plotted in Figure 1. In real-world data analysis, XgBoost-based PS matching resulted in the best covariate balance, with the lowest ASMD leading to all covariates with ASMD *<* 0.1 post-matching. On the other hand, PS matching based on LASSO, reference method and MLP resulted in a comparatively poorer balance, with certain covariates displaying an ASMD exceeding 0.1 after matching. Details of imbalanced covariates are available in Supplementary Material. In the plasmode simulation, the ASMD results for all methods showed a similar level of balance, with XgBoost and LASSO being able to achieve the lowest ASMD.

**TABLE 1:** Covariate balance for real-world data and plasmode simulation: before and after PS matching scatterplot of absolute standardised differences.

	ASMD - PS plasmode	ASMD - PS real-world data
Reference method	0.1032 (0.1020, 0.1045)	0.0394
LASSO	0.0991 (0.0978, 0.1003)	0.0167
XgBoost	0.0990 (0.0968, 0.1011)	0.0150
MLP	0.1010 (0.0992, 0.1027)	0.0480

#### 3.2.2 Treatment effect estimation after propensity score matching - negative control outcome analysis in real-world data

All tested methods showed some residual bias, with negative control outcomes coverage of the null consistently below 70% for all tested methods (Table 2).

**TABLE 2:** Negative control outcome analysis for propensity score matching results.

PS estimation method	Coverage (%)	RMSE
Reference	62.3	0.1766
XgBoost	63.8	0.1741
LASSO	57.1	0.1709
MLP	53.3	0.1763

**FIGURE 2:**
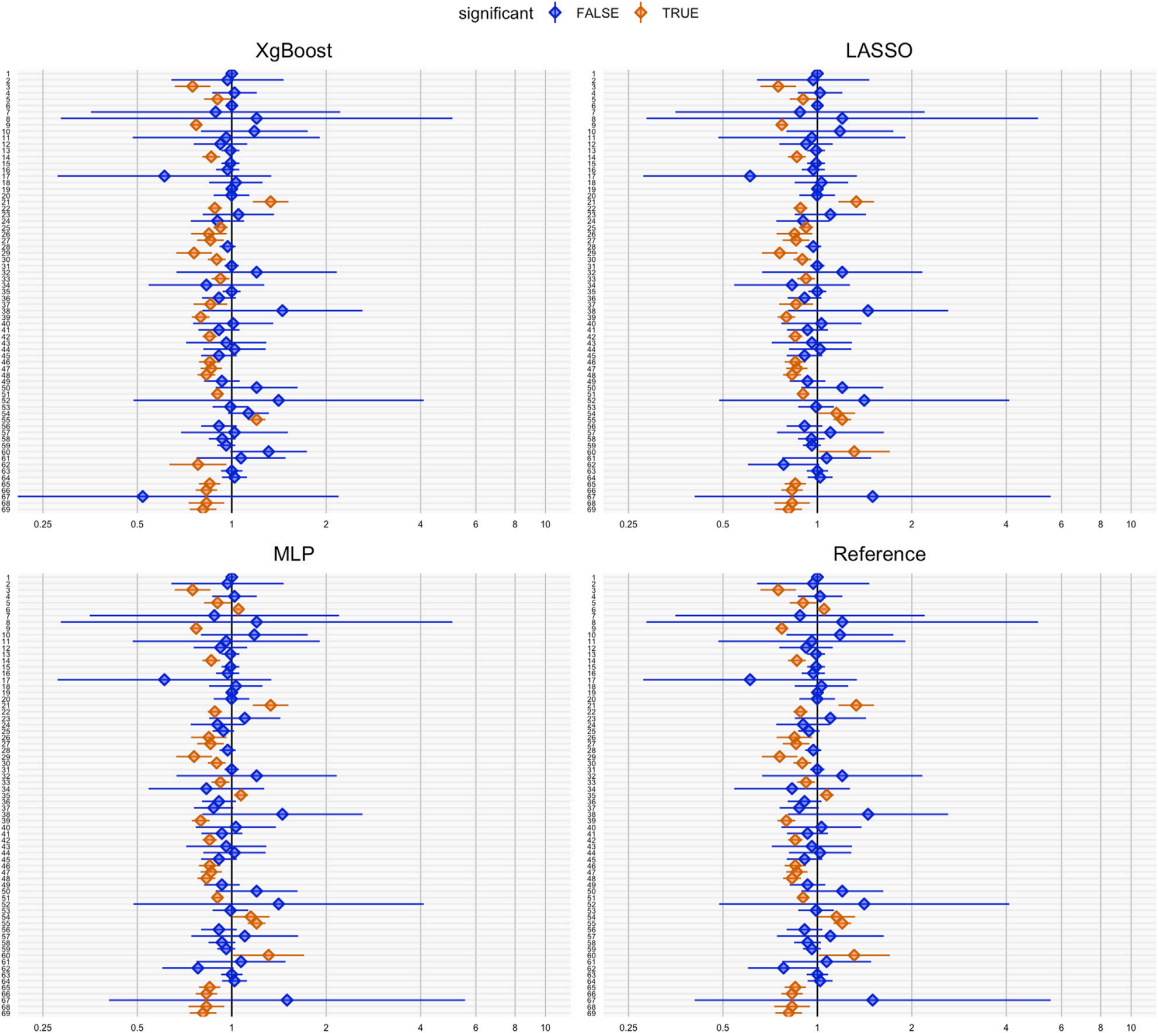
Coverage plots of negative control outcomes for each propensity score method: XgBoost, LASSO, MLP and reference. Each dot represents the hazard ratio estimation for a negative control outcome. Hazard ratios significantly different from 1 (based on a significance level of 0.05) are highlighted in orange, indicating potential bias or confounding, while non-significant results are shown in blue. The horizontal lines depict the confidence intervals for each hazard ratio.

Figure 2 illustrates effect estimates and 95% confidence intervals for each negative control outcome after PS matching. The *y*-axis (ID 1-69) represents all tested negative control outcomes introduced above. The hazard ratio estimates, including the null effect of 1, indicate coverage of negative control outcomes. XgBoost achieved the highest coverage for negative control outcomes (63.8%), while MLP resulted in the lowest (53.3%). For RMSE in hazard ratio estimation, all methods had similar values (Table 2).

#### 3.2.3 Treatment effect estimation after propensity score matching - plasmode simulation

Table 3 presents relative bias for the estimated treatment effect after PS matching using the four methods in the plasmode simulation. XgBoost showed the lowest average relative bias (0.5473 (0.4802, 0.6144)), consistent with the best covariate balance. Although confidence intervals overlapped for all methods, the results aligned with real-world data analysis findings, pointing to XgBoost-based PS as the best-performing method.

**TABLE 3:** Propensity score method: treatment effect estimation relative bias with 95% Confidence Intervals for the plasmode experiment.

PS estimation method	Relative bias - plasmode simulation
Reference	0.5593 (0.4950, 0.6237)
LASSO	0.5709 (0.5037, 0.6382)
XgBoost	0.5473 (0.4802, 0.6144)
MLP	0.5585 (0.4905, 0.6264)

### 3.3 Comparison of disease risk scores and propensity scores results

#### 3.3.1 Propensity scores vs. disease risk scores covariate balance in real-world data and plasmode simulation

**FIGURE 3:**
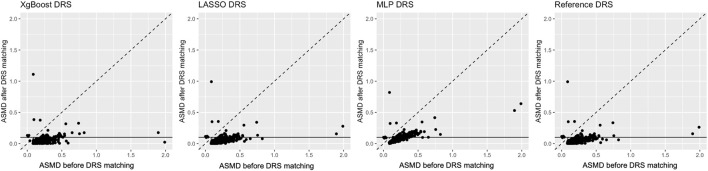
Visualisation for real-world data analysis: Each panel shows the average standardised absolute mean difference (ASMD) before (*X*-axis) and after disease risk score matching (*Y*-axis). The horizontal line indicates the threshold of 0.1, below which the ASMD after matching is considered acceptable.

Covariate balance post-DRS matching was evaluated in both real-world and plasmode simulation data. Figure 3 depicts the pre- and post-matching ASMD for each covariate in real-world data analysis. The pre-matching ASMD was 0.1977 for real-world data and 0.1943 (0.1934, 0.1952) for plasmode simulation. Figure 3 shows ASMD before and after DRS matching. For all estimation methods, there were covariates with ASMD over 0.1 after DRS matching. Details of these imbalanced covariates and average ASMD after DRS matching can be found in Supplementary Material.

The ASMD values after matching for DRS in plasmode simulation were 0.0973 (0.0961, 0.0986), 0.1223 (0.1192, 0.1253), 0.1167 (0.1140, 0.1195) and 0.1007 (0.0988, 0.1026) for the reference, LASSO, XgBoost and MLP method respectively, in real-world data analysis the ASMD after DRS matching were 0.0426, 0.0442, 0.0430 and 0.0703 for the reference, LASSO, XgBoost and MLP method respectively.

Notably, DRS matching, regardless of the estimation method, resulted in a similar or stronger covariate imbalance, compared to PS. The reference method achieved the lowest ASMD in both real-world data and plasmode simulation analyses.

**FIGURE 4:**
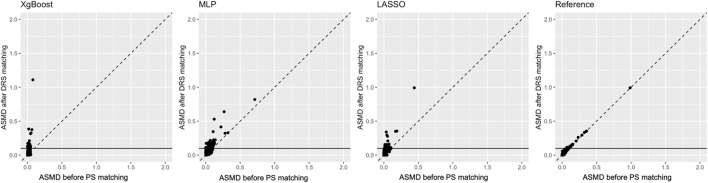
Comparison of propensity score and disease risk score matching methods: average standardised absolute mean difference (ASMD) after propensity score matching (*X*-axis) versus ASMD after disease risk score matching (*Y*-axis). The horizontal line indicates the threshold for acceptable balance (ASMD <0.1).

The plot comparing ASMD values after DRS matching for each covariate against those after PS matching is shown in Figure 4. Matched on PS, particularly estimated by XgBoost and LASSO, achieved better covariate balance than DRS matching with the same methods. However, the performance of PS estimated by the reference method was similar to that of DRS.

#### 3.3.2 Propensity scores vs. disease risk scores treatment effect estimation after matching negative control outcome analysis in real-world data

**FIGURE 5:**
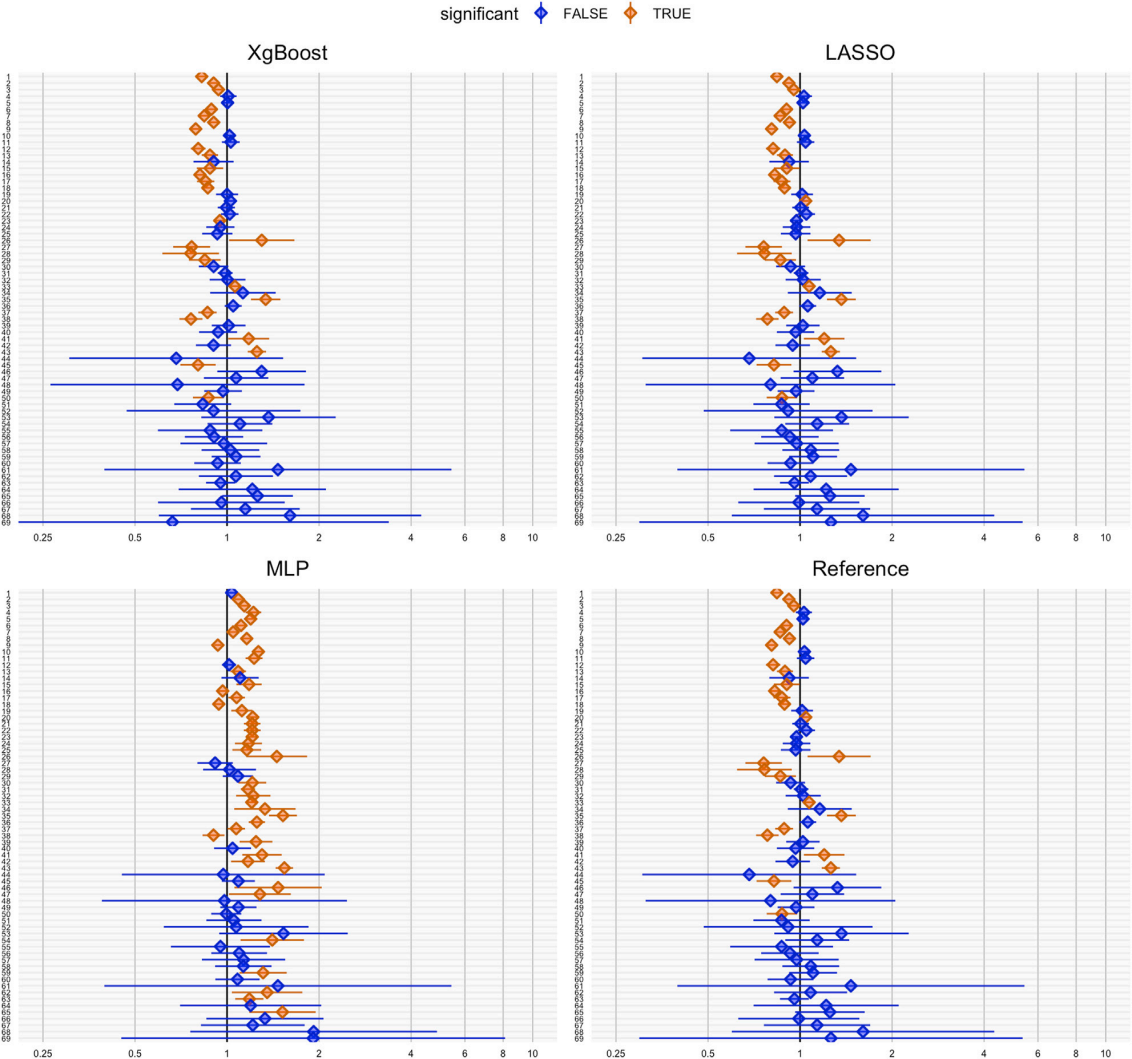
Coverage plots for negative control outcomes using XgBoost, LASSO, MLP, and Reference. Each dot represents a hazard ratio estimate for a negative control outcome. Significant deviations from one (*p* < 0.05) are shown in orange, indicating potential bias or confounding, while non-significant results are shown in blue. Horizontal lines depict confidence intervals for each hazard ratio.

Figure 5 illustrates negative control outcomes for each DRS estimation method. The wider blue confidence intervals, show hazard ratio estimations with lower statistical power compared to PS matching (Figure 2). Despite higher coverages, DRS matching led to higher RMSEs for negative control outcome hazard ratio estimations.

The coverage for negative control outcome hazard ratio estimation and RMSE for hazard ratio estimation after DRS matching is shown in Table 4. All of the DRS estimation methods showed residual bias, as evidenced by the observed coverage for all negative control outcomes being below 70%. In contrast to the PS coverage when evaluating real-world data, both the MLP and XgBoost methods yielded worse results when used for DRS estimation, with a reduced coverage of only 37.7% for MLP-based DRS, and 62.3% for XgBoost-based DRS matching. Conversely, the LASSO method had an increase in coverage, from 57.1% to 63.8%, thereby attaining the highest coverage.

**TABLE 4:** Negative control outcome analysis for disease risk score matching results, and compared to PS matching.

Estimation method	Coverage (DRS) (%)	Coverage (PS) (%)	RMSE (DRS)	RMSE (PS)
Reference	62.3	62.3	0.1766	0.1766
XgBoost	62.3	63.8	0.1809	0.1741
LASSO	62.3	57.1	0.1766	0.1709
MLP	37.7	53.3	0.2783	0.1763

The LASSO method excelled in hazard ratio estimation, yielding the lowest RMSE of 0.1766. For negative control outcomes, all ML methods, except the reference method, exhibited increased RMSEs with DRS matching compared with PS matching. This emphasises that, in ML-based PS and DRS estimation, PS matching consistently resulted in reduced RMSE for treatment effects relative to DRS, consistent with earlier observations on covariate balance.

#### 3.3.3 Propensity scores vs. disease risk scores treatment effect estimation after matching - plasmode simulation

Relative bias after DRS matching using four DRS estimation methods is shown in Table 5. The reference method and the MLP method led to the lowest bias. In contrast, LASSO and XgBoost registered slightly higher average relative biases, at 0.7142 and 0.7091 respectively. In the comparison between PS and DRS, the relative bias observed from PS matching was consistently lower than that from DRS matching.

**TABLE 5:** Disease risk score versus propensity score method: treatment effect estimation relative bias with 95% confidence intervals for the plasmode experiment.

	DRS relative bias	PS relative bias
Reference	0.6833 (0.6130, 0.7537)	0.5593 (0.4950, 0.6237)
LASSO	0.7142 (0.6433, 0.7852)	0.5709 (0.5037, 0.6382)
XgBoost	0.7091 (0.6383, 0.7798)	0.5473 (0.4802, 0.6144)
MLP	0.7072 (0.6371, 0.7773)	0.5585 (0.4905, 0.6264)

## 4 Discussion

This study provides key insights into ML-based PS and DRS estimation. Beyond treatment effect estimation bias, we also used outcome-independent metrics like ASMD to provide a more objective assessment of model effectiveness. This aligns with the literature’s emphasis on the importance of not relying solely on outcome-dependent metrics like bias (Tian et al., 2018). Furthermore, while external validation would further strengthen these findings, the internal validation approach provides valuable insights into the relative performance of the models. We used 10-fold cross-validation to tune hyperparameters and assess model performance using Brier score loss as an out-of-sample metric. This approach helps ensure the generalisability of the models. While external validation would provide additional robustness, the 10-fold cross-validation serves as a rigorous internal validation method, preventing overfitting and offering valuable insights into the relative performance of the methods. Future work could explore external validation if suitable data become available to further strengthen the findings.

To our knowledge, no studies have systematically compared PS versus DRS using ML methods for estimating treatment effects. Most research has focused on regression methods, underlining the novelty of this study and emphasising the need for further exploration of ML-based methods in PS and DRS estimation.

Each ML method selected in this study carries inherent assumptions that influence performance. LASSO assumes linearity and sparsity, making it less effective for non-linear relationships, while XgBoost’s additive decision tree structure handles non-linear interactions and imbalanced data better (Chen and Guestrin, 2016). MLP, though powerful for complex non-linear modelling, requires balanced data and large sample sizes, making it more sensitive to the imbalanced data in this study (Huang et al., 2022). These assumptions help explain the superior performance of XgBoost in both PS and DRS estimation, particularly in handling nonlinear real-world data, while LASSO’s regularisation proved effective in managing less complicated simulated data. Moreover, after hyperparameter tuning, XgBoost demonstrated superior performance in PS estimation, exhibiting the lowest ASMD and relative bias for treatment effect estimation, consistent with prior research on using ASMD for hyperparameter selection (McCaffrey et al., 2004; Cannas and Arpino, 2019). However, XgBoost’s efficacy in DRS estimation was less pronounced, possibly due to imbalanced targets under the rare event data. Future research could explore methods like synthetic minority oversampling method (Rivera et al., 2014) to improve ML performance in imbalanced data scenarios.

Our findings suggest that XgBoost and LASSO can estimate PS comparably or better than logistic regression models based on prior confounder knowledge. These methods are scalable for large data, especially when analysing multiple treatments or outcomes. Additionally, evolutionary computation methods like genetic algorithms and harmony search (Tuo et al., 2022) show potential for complementing the ML methods explored here, offering future directions for refining PS and DRS estimation. Moreover, ensemble approaches like Super Learner, which combine multiple algorithms for robust predictions, present promising avenues for further enhancing model accuracy and generalisability (Pirracchio et al., 2015).

Despite these promising results, the study has limitations. The low coverage in 95% confidence intervals for negative control outcomes likely extends beyond confounding bias and may involve selection or information bias. In addition, the absence of external validation and potential unmeasured confounders restrict the generalisability of these findings. However, incorporating RMSE for negative control outcomes provided a more comprehensive evaluation, and the plasmode simulation results further validated these findings.

In real-world data analyses, ML methods like XgBoost and MLP may face challenges in widespread implementation due to the need for expert input in tuning and interpretation. XgBoost, while highly effective for structured data, is computationally intensive, particularly when optimising numerous hyperparameters for large data (Chen and Guestrin, 2016). MLP, though capable of modelling complex non-linearities, requires careful tuning to handle imbalanced data effectively and to prevent biased estimation (Huang et al., 2022). In contrast, LASSO is faster to tune and computationally efficient, making it easier to apply in large-scale clinical data. However, it is less capable of capturing complex relationships between covariates, making it more suitable for simpler, linear models. Furthermore, ethical considerations, such as model transparency and algorithmic bias, must also be addressed, as they have implications for decision-making and patient outcomes (Chin et al., 2023). As ML methods continue to evolve, the consideration of these factors will be crucial for their successful application into clinical practice.

## 5 Conclusion

ML methods with hyperparameter tuning and the logistic regression model with pre-selected covariates were tested on real-world data and plasmode simulation data for PS and DRS estimation to assess treatment effects. ML methods, particularly XgBoost, demonstrated superior covariate balance and less treatment effect estimate bias compared to traditional logistic regression. ML-based PS methods performed better than DRS methods, highlighting the need for future research on their application in diverse scenarios.

## Data Availability

The datasets presented in this article are not readily available because this study is based on patient-level confidential data, however, the code to apply the methods is available in Github, provided in Supplementary Material. Requests to access the datasets should be directed to yuchen.guo@ndorms.ox.ac.uk.
