# Supplementary material for: Machine learning methods for propensity and disease risk score estimation in high-dimensional data: a plasmode simulation and real-world data cohort analysis
Source: Front Pharmacol. 2024 Oct 28;15:1395707. doi: 10.3389/fphar.2024.1395707 (PMC11551032; doi:10.3389/fphar.2024.1395707)
Supplement: Supplementary file 1 [file DataSheet1.PDF]

Supplementary Material

1 REAL-WORLD DATA SETTINGS

1.1 Study Design

Figure S1 illustrates the process and timeline.

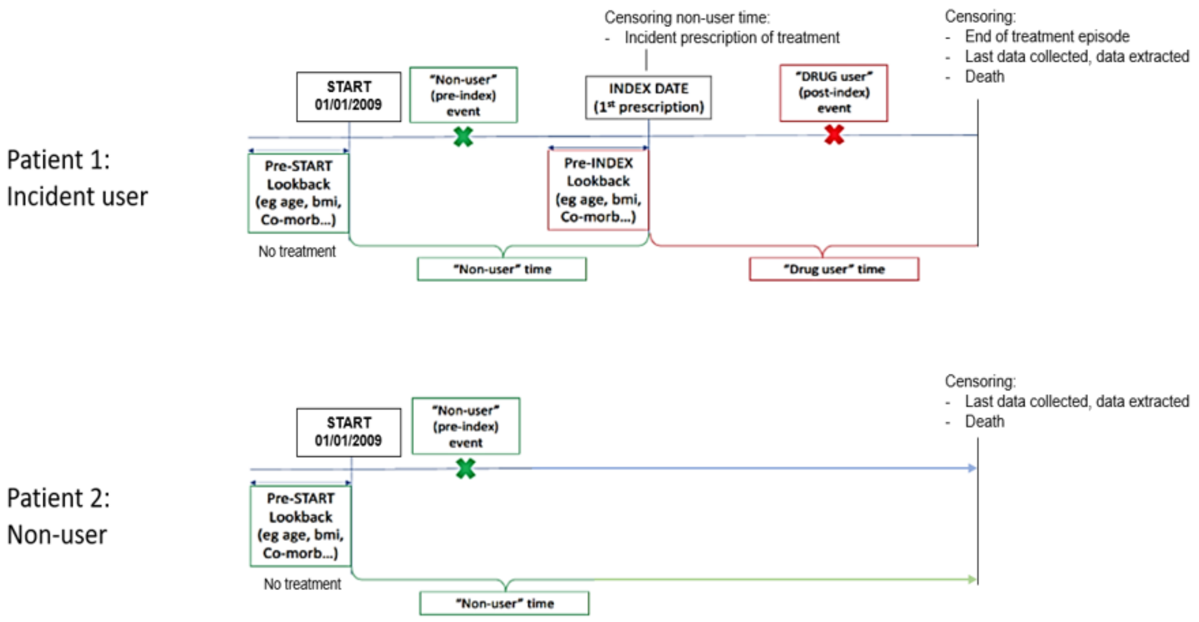

Figure S1. Study design demonstration: user versus non-user definition

Follow-up ended at the earliest occurrence of any of these events: a participant's transfer out of the study (due to relocation, etc.), the final data collection date from the practice, or the participant's death. Censoring differed based on the treatment status:

- 'Non-users' were censored on the day of treatment initiation, which is the index date when they transitioned to 'drug users'. In cases where no treatment was initiated, censoring occurred at the earliest of the follow-up occurrence criteria outlined above.
- 'Drug users' were censored at the end of the treatment period that began on the index date, based on the earliest of the follow-up occurrence criteria outlined above, if no further continuous antihypertensive therapy episodes were recorded.

1.2 Covariates

We created 637 covariates, following OHDSI covariates OMOP CDM Version 5 format, using the FeatureExtraction package in R, with the following setting:

- useDemographicsGender = TRUE

- useDemographicsAge = TRUE
- useDrugEraMediumTerm = TRUE
- useDrugEraShortTerm = TRUE
- useConditionOccurrenceAnyTimePrior = TRUE
- endDays = -1
- longTermStartDays = -365
- mediumTermStartDays = -180
- shortTermStartDays = -30
- useVisitCountShortTerm = TRUE

Then we chose covariates with greater than 0.004 prevalence in data, hence generated 637 covariates: age, gender = MALE, gender = FEMALE, **drug era observed or not during day -180 through -1 days relative to index** for following drugs: erythromycin, acetaminophen, influenza virus vaccine & unspecified formulation, prednisolone, lidocaine, clarithromycin, clavulanate, folic acid, lactulose, calcium, metformin, naproxen, polawax polysorbate, alginate, alendronate, floxacillin, fusidate, diclofenac, dihydrocodeine, levothyroxine, sodium, salicylic acid, ibuprofen, loperamide, sennoside B, clotrimazole, Novel influenza-H1N1-09, injectable, tramadol, bicarbonate ion, lanolin, petrolatum, amoxicillin, doxycycline, nitrofurantoin, salmeterol, isopropyl myristate, polyethylene glycol 3350, psyllium, ranitidine, hypromellose, betamethasone, beclomethasone, finasteride, temazepam, citalopram, benzalkonium, diazepam, cephalexin, fluoxetine, ferrous sulfate, warfarin, miconazole, codeine, chlorhexidine, estradiol, tiotropium, tamsulosin, Influenza, seasonal, injectable, atorvastatin, amitriptyline, mometasone, nystatin, latanoprost, dexamethasone, albuterol, gliclazide, timolol, pneumococcal vaccine, unspecified formulation, neomycin, prochlorperazine, loratadine, alginic acid, chloramphenicol, paraffin, quinine, betahistine, hydroxocobalamin, zopiclone, potassium, fluticasone, cholecalciferol, mebeverine, trimethoprim, cetirizine, aspirin, omeprazole, mineral oil, hydrocortisone, carbomer homopolymer type c, ciprofloxacin, lansoprazole, phenoxyethanol, zinc oxide, oxytetracycline, simvastatin, clobetasone.

Condition occurrence observed or not, any time prior through -1 days relative to index, for the following events/conditions: At risk of osteoporosis, Patient's condition improved, Elbow joint pain, Palpitations, Skin lesion, Respiratory tract infection, Muscle pain, Osteoarthritis of hip, Candidiasis of mouth, Pain in calf, Pyoderma, Exercise physically impossible, Swollen ankle region, Mild chronic obstructive pulmonary disease, Urine protein test negative, Sinus headache, Dyspnea, Backache, Tinea pedis, Onychomycosis due to dermatophyte, Pain in limb, Ca cervix screening normal, Cyst of skin, Bunion, Cervical smear result, Fracture of humerus, Abdomen examined - NAD, Acute upper respiratory infection, Tight chest, Menopausal flushing, Acute tracheitis, Seborrheic dermatitis, Fatigue, Blurred vision, Anxiety disorder, Mammography normal, Injury of ankle, Influenza-like illness, O/E - left dorsalis pedis normal, Multiple joint pain, Bilateral cataracts, Acute laryngitis, Foot pain, Polymyalgia rheumatica, Disorder of eye, O/E - vibration sense normal, Lower respiratory tract infection, Ear symptom, Altered bowel function, Fully mobile, Drug overdose, Ganglion cyst, Sensorineural hearing loss, Sprain of spinal ligament, Chronic rhinitis, Mixed anxiety and depressive disorder, Muscle strain, Dry eyes, Hand pain, Depressive disorder, Productive cough -green sputum, Impacted cerumen, Acid reflux, Alzheimer's disease, Pruritus ani, Urinary symptoms, Urinary tract infectious disease, Uterine leiomyoma, O/E - BP reading normal, Diabetes mellitus, Uterovaginal prolapse, Allergic disposition, Indigestion, Diverticular disease, Vertigo, Insect bite - wound, Upper respiratory infection, Hemorrhoids, Sensation of blocked ear, Fracture of radius, Diarrhea symptom, Injury of finger, Renal colic, Intertrigo, Cyst of epididymis, Gastroenteritis,

Breast lump, Medial epicondylitis, O/E - visual acuity R-eye, No abnormality detected - examination result, Cough, Musculoskeletal pain, Iron deficiency anemia, Pain in testicle, Spondylosis, Cerebrovascular accident, Sprains and strains of joints and adjacent muscles, Headache, Reactive depression, Esophageal reflux finding, Dysfunction of eustachian tube, Pain in toe, Dental abscess, Angina pectoris, Clinical history and observation findings, Chronic obstructive lung disease, Hearing difficulty, O/E - chest examination normal, Pharyngitis, O/E - Vibration sense of right foot normal, Corn - lesion, Glaucoma, Osteoarthritis, C/O - loin pain, Conjunctivitis, Pain in thumb, Presbycusis, Inguinal pain, Bladder: fully continent, Tingling of skin, Vasovagal syncope, Sprain of sacroiliac ligament, Supraspinatus tendinitis, O/E - vaginal discharge, Benign paroxysmal positional vertigo or nystagmus, 10g monofilament sensation L foot normal, Orthostatic hypotension, Injury of knee, Pain in face, Asthma never causes daytime symptoms, Ulcer of duodenum, Serum cholesterol raised, Clouded consciousness, 10g monofilament sensation R foot normal, Actinic keratosis, Varicose eczema, Acute respiratory infections, Standard chest X-ray normal, Acute stress disorder, Tinnitus, Vomiting, Arthritis, Wheezing symptom, Inguinal hernia, O/E - wound healing, Recurrent urinary tract infection, Inflammatory dermatosis, Knee pain, Pruritus of skin, Hemoptysis, Tendinitis, Plane wart, O/E - pulse rate, Generalized aches and pains, Labyrinthitis, Gallstone, Dizziness, Verruca plantaris, Cervical smear - inadequate specimen, Low back pain, Cystitis, Type 2 diabetes mellitus, Traumatic AND/OR non-traumatic injury, Cervical smear - borderline changes, Atopic dermatitis, O/E - mental state, Pruritus of vulva, Verruca vulgaris, Amnesia, Skin irritation, Chronic catarrhal rhinitis, Enjoys light exercise, Senile hyperkeratosis, Sciatica, Edema, Vitreous floaters, Pain in throat, Subconjunctival hemorrhage, Trochanteric bursitis, Gastroesophageal reflux disease, Diarrhea, Atrophic vaginitis, Constipation, Seborrheic dermatitis of scalp, Urinary incontinence, Dupuytren's contracture, Menopause present, C/O - cough, Enjoys moderate exercise, Injury of head, Malaise, Asthma resolved, Hiatal hernia, Herpes labialis, Carpal tunnel syndrome, Olecranon bursitis, Hypothyroidism, Vaginal discharge symptom, Impetigo, Pain in lower limb, Lymphadenopathy, Gastritis, Vaginal bleeding, On examination - peripheral pulses left leg, Contusion with intact skin, O/E - wax in auditory canal, Cervical spondylosis without myelopathy, Rectal hemorrhage, O/E - ear, Worried, Chronic sinusitis, Dermal mycosis, Hyperlipidemia, C/O - wax in ear, Plantar fasciitis, O/E - hearing tested-8th nerve, Cervical smear - negative, Pain in lumbar spine, Tired all the time, Respiratory symptom, Essential hypertension, Has an itchy eye, Exercise grading, Chronic kidney disease stage 3, Ocular hypertension, Neck pain, Productive cough, Insomnia, Bloating symptom, Hypertensive disorder, Migraine, Rib pain, Eczema, Gastro-esophageal reflux disease with esophagitis, Melanocytic nevus of skin, Acute pharyngitis, Fracture of bone, Pain of breast, Acute tonsillitis, Fracture of neck of femur, O/E - right foot pulses present, Gout, Epilepsy, Pins and needles, Asthma not disturbing sleep, Degeneration of macula and posterior pole, Ingrowing nail, Delivery normal, Acute bronchitis, Obesity, Xerostomia, Acute exacerbation of chronic obstructive airways disease, Injury of foot, Degenerative joint disease involving multiple joints, Furuncle, Postoperative wound infection, Heartburn, Chalazion, Influenza, Esophagitis, Patient condition unchanged, C/O - a headache, O/E - visual acuity L-eye, Lipoma, Abdominal discomfort, Papilloma of skin, Pure hypercholesterolemia, Otalgia, O/E - pulse rhythm regular, Red eye, C/O: a rash, Otitis media, Thyrotoxicosis, Phlebitis and thrombophlebitis, Heel pain, Wheezing, Medical Research Council Dyspnoea scale grade 3, Giddiness, C/O: a pain, Deafness symptom, Deep venous thrombosis, Medical Research Council Dyspnoea scale grade 1, New medication added, Medication decreased, Arthropathy, General symptom, C/O - postnasal drip, Prostatism, Lateral epicondylitis, Basal cell carcinoma of skin, Pain in eye, Dysuria, Numbness, Viral disease, Atopic conjunctivitis, O/E - foot, Mass of skin, Cerebrovascular disease, Dry cough, Retention of urine, Common cold, Breast neoplasm screening normal, Asthma, Urgent desire to urinate, Cramp in lower limb, Solitary cyst of breast, Hoarse, Irritable bowel syndrome, Otitis externa,

Lumbar spondylosis, Pleuritic pain, C/O - catarrh, Joint pain, Acute myocardial infarction, Shoulder pain, Nasal congestion, Ankle pain, O/E - BP reading raised, Rheumatoid arthritis, Nocturia, Allergic rhinitis due to pollen, Transient cerebral ischemia, Hearing loss, Cataract, Achilles tendinitis, Infective pneumonia, Dyspnea on exertion, Panic attack, Gastroduodenitis, Ischemic heart disease, Ulcer of lower extremity, Genuine stress incontinence, Visual symptoms, Syncope, Rosacea, Pain, Hip pain, O/E - hernia, Acute exacerbation of asthma, Paronychia, Abdominal pain, Acute conjunctivitis, Muscle weakness, Acquired hallux valgus, Acquired hypothyroidism, Malignant neoplasm of female breast, Malignant tumor of prostate, Peripheral edema, Foot problem, O/E - right dorsalis pedis normal, Urticaria, Atrial fibrillation, Acquired trigger finger, Pulmonary embolism, Epidermal nevus, C/O: itching, C/O - feeling depressed, Candidiasis of vagina, Synovitis and tenosynovitis, External hordeolum, O/E - left foot pulses present, Osteoporosis, C/O: a swelling, Acute lower respiratory tract infection, Infected insect bite, Bowels: fully continent, Abdominal colic, O/E - Vibration sense of left foot normal, Asthma not limiting activities, Wax in ear canal, Bleeding from nose, Eye symptom, Wrist joint pain, Idiopathic detrusor overactivity, O/E - blood pressure reading, Cramp in lower leg associated with rest, Collapse, On examination - general breast examination - no abnormality detected, Breast lump symptom, Sprain of ankle, Closed Colles' fracture, Epigastric pain, Infection of sebaceous cyst, Rotator cuff syndrome, External hemorrhoids without complication, Candidiasis, Bronchitis, Vitreous detachment, Injury of wrist, Whiplash injury to neck, Paresthesia, Vulval irritation, Eruption, Avoids even trivial exercise, Skin tag, Musculoskeletal chest pain, Infective otitis externa, Sore throat symptom, Impotence, C/O - low back pain, Anxiety state, Asthma daytime symptoms, Tension-type headache, Onychomycosis, Increased frequency of urination, Musculoskeletal and connective tissue disorder, Sinusitis, Cervical spondylosis, Diarrhea and vomiting, symptom, Polyp of nasal cavity and/or nasal sinus, European, O/E - BP borderline raised, Chronic kidney disease stage 2, Epidermoid cyst of skin, Ulcer of mouth, Dermatophytosis, Patient's condition worsened, Osteopenia, Problem knee, Metatarsalgia, Depressed mood, Dyssomnia, Urine glucose test negative, Miscarriage, Nausea, On examination - left leg pulses all present, O/E - vaginal examination, Cystocele, Infection of skin and/or subcutaneous tissue, Postmenopausal bleeding, O/E - itchy rash, Lightheadedness, Folliculitis, Patient feels well, Tremor, Benign prostatic hyperplasia, Nasal discharge, Psoriasis, Pain in upper limb, Tear film insufficiency, O/E - dry skin, Fracture of ankle, Dysphagia, Adhesive capsulitis of shoulder, Elevated blood pressure, O/E - edema of ankles, Anxiety, Anemia, Disorder of skin and/or subcutaneous tissue, Medication increased, Medical Research Council Dyspnoea scale grade 2, Allergic rhinitis, Osteoarthritis of knee, Nasal obstruction, Herpes zoster, Contact dermatitis, On examination - right-leg pulses all present, Spontaneous bruising, Visual disturbance, Nocturnal cough / wheeze, Multiple symptoms, Urinalysis = no abnormality, Sore mouth, Cellulitis, Chest pain, Blood in urine, Neuralgia, Restless legs, O/E - allergic rash, Menorrhagia, Blepharitis, Complaining of insomnia, Lethargy, O/E - a lump, Acute sinusitis, Varicose veins of lower extremity, Earache symptoms, Nasal symptom, White, On examination - peripheral pulses right leg, Laceration - injury, Diverticulitis, Tinea cruris, Anal fissure.

Drug era observed or not during day -30 through -1 days relative to index, for the following drugs: alginic acid, fluticasone, levothyroxine, timolol, quinine, calcium, amitriptyline, tamsulosin, acetaminophen, hypromellose, diclofenac, tiotropium, paraffin, temazepam, diazepam, omeprazole, folic acid, ranitidine, dihydrocodeine, finasteride, latanoprost, metformin, Novel influenza-H1N1-09, injectable, aspirin, influenza virus vaccine, unspecified formulation, alendronate, warfarin, citalopram, amoxicillin, tramadol, prednisolone, betamethasone, hydrocortisone, petrolatum, mometasone, atorvastatin, ibuprofen, sennoside B, psyllium, simvastatin, potassium, polyethylene glycol 3350, salmeterol, lactulose, sodium, beclomethasone, codeine, albuterol, zopiclone, bicarbonate ion, mineral oil, carbomer homopolymer type c, cholecalciferol, lansoprazole

The identification of fracture was done using code in JSON, can be found here: GitHub.

2 NEGATIVE CONTROL OUTCOMES SETTINGS

The following 69 negative control outcomes were selected: Onychomycosis due to dermatophyte, Ingrowing nail, Alzheimer's disease, Degeneration of cervical intervertebral disc, Backache, Chronic kidney disease stage 3, Acute upper respiratory infection, Lumbosacral spondylosis without myelopathy, Cardiomegaly, Abdominal pain, Asthma, Iron deficiency anemia, Raised prostate specific antigen, Diverticular disease of colon, Diarrhea, Atrial fibrillation, Disorder of nail, Shoulder joint pain, Hematuria syndrome, Urinary tract infectious disease, Muscle weakness, Acute exacerbation of chronic obstructive airways disease, Ulcer of foot, Renal disorder due to type 2 diabetes mellitus, Low back pain, Constipation, Fever, Benign neoplasm of colon, Atelectasis, Osteoarthritis, Hypothyroidism, Sepsis, Pain in thoracic spine, Nonexudative age-related macular degeneration, Cataract, Anemia, Dysphagia, Degenerative joint disease involving multiple joints, Generalized abdominal pain, Chest pain, Actinic keratosis, Gastroesophageal reflux disease, Walking disability, Organic mental disorder, Osteoporosis, Rheumatoid arthritis, Impaired fasting glycemia, Arthralgia of the pelvic region and thigh, Localized, primary osteoarthritis, Anxiety disorder, Allergic rhinitis, Exudative age-related macular degeneration, Disorder of lung, Malignant lymphoma, Acute sinusitis, Multiple myeloma, Atrial flutter, Spinal stenosis of lumbar region, Idiopathic peripheral neuropathy, Muscle pain, Cervical spondylosis without myelopathy, Seizure, Type 1 diabetes mellitus, Disorder of bone and articular cartilage, Pain in limb, Arthropathy, Tear film insufficiency, Primary open angle glaucoma.

3 PLASMODE SIMULATION SETTINGS

A demonstration of this process is shown in Figure S2.

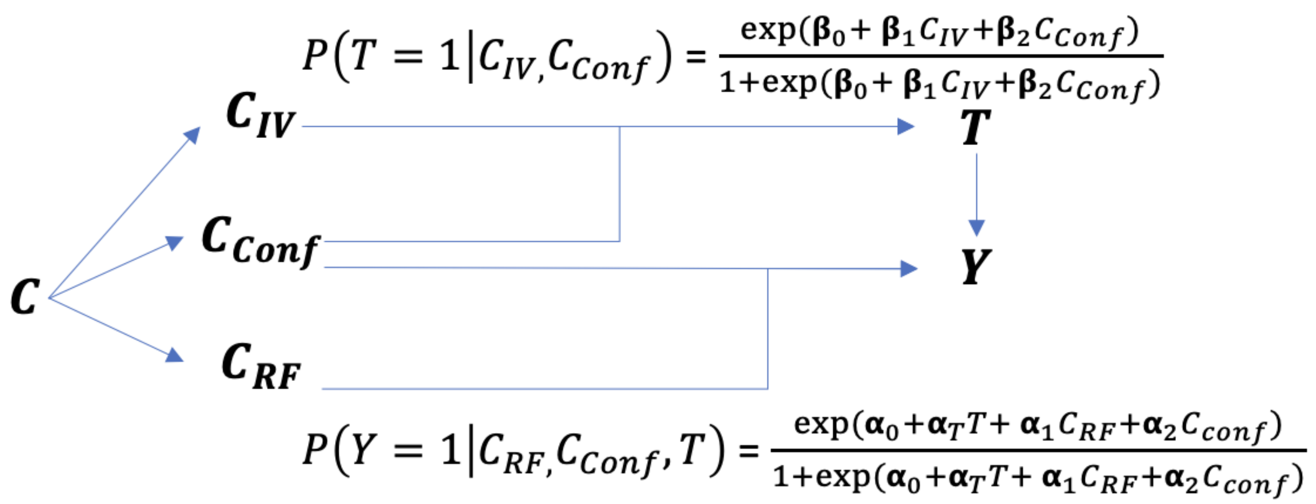

Figure S2. Demonstration of plasmode simulation process with instrumental variables, risk factors and confounders

Denote all covariates in the clinical data as C, confounders (variables associated with both treatment and outcome) as C_{Conf}, instrumental variables (variables associated only with the treatment) as C_{IV}, and risk factors (variables associated only with the outcome) as C_{RF}. The coefficients vector used to generate

synthetic treatment denote $\boldsymbol{\beta}$. Specifically, $\boldsymbol{\beta_1}$ and $\boldsymbol{\beta_2}$ denote The coefficient vectors for all covariates included in treatment generation. β_0 represents the intercept coefficient, which was used to make the treatment prevalence of plasmode simulated data the same as the original clinical data (0.2588). In the outcome generation process, α_T denotes the coefficient for treatment effect, and α_0 denotes the intercept coefficient, which was used to adjust the outcome risk of plasmode simulated data the same as the original clinical data (0.0075). $\boldsymbol{\alpha_1}$ and $\boldsymbol{\alpha_2}$ denote the coefficient vectors for all covariates included in the outcome generation process.

1. Specify which variables out all covariates $\boldsymbol{C}$ in dataset: instrumental variables $\boldsymbol{C_{IV}}$, risk factors $\boldsymbol{C_{RF}}$, and confounders $\boldsymbol{C_{Conf}}$
2. Coefficients β_0, β_1 and β_2 were obtained from fitting a binary logistic propensity score model on the real-world data, where the response variable was T and the explanatory variables were in C_{IV} and C_{Conf}. Then generate treatment with the probability $P(T = 1|\boldsymbol{C_{IV}}, \boldsymbol{C_{Conf}})$ from Bernoulli distributions:

$$P(T = 1|\boldsymbol{C_{IV}}, \boldsymbol{C_{Conf}}) = \frac{exp(\beta_0 + \boldsymbol{\beta_1 C_{IV}} + \boldsymbol{\beta_2 C_{Conf}})}{1 + exp(\beta_0 + \boldsymbol{\beta_1 C_{IV}} + \boldsymbol{\beta_2 C_{Conf}})}$$

3. Coefficients α_0,α_t, α_1 and α_2 were obtained from fitting a binary logistic propensity score model on the real-world data, where the response variable was Y and the explanatory variables were T, C_{RF} and C_{Conf}. Then the true treatment effect α_t was modified to the user-defined value (log(1.5)). Then generate the outcome with the probability from Bernoulli distributions:

$$P(Y = 1|\boldsymbol{C_{RF}}, \boldsymbol{C_{Conf}}) = \frac{exp(\alpha_0 + \alpha_T T + \boldsymbol{\alpha_1 C_{RF}} + \boldsymbol{\alpha_2 C_{Conf}})}{1 + exp(\alpha_0 + \alpha_T T + \boldsymbol{\alpha_1 C_{RF}} + \boldsymbol{\alpha_2 C_{Conf}})}$$

In our plasmode simulation, for computation cost reason for the following hyperparameter tuning process, we reduced the number of covariates down to 90 (50 confounders, 20 risk factors and 20 instrumental variables), and the number of observations to 10000. Covariates used to generate plasmode simulation data are: drug era observed or not during day -180 through -1 days relative to index or not, for the following drugs: salmeterol, lansoprazole, hydroxocobalamin, alendronate, polyethylene glycol 3350, Influenza, seasonal, injectable, ibuprofen, sodium, floxacillin, acetaminophen, doxycycline, atorvastatin, beclomethasone, carbomer homopolymer type c, petrolatum, aspirin, ciprofloxacin, warfarin, mometasone, influenza virus vaccine, unspecified formulation, hydrocortisone, betahistine, cholecalciferol, tamsulosin, simvastatin, polawax polysorbate, hypromellose, nitrofurantoin, mebeverine, quinine, Novel influenza-H1N1-09, injectable, temazepam, prednisolone, codeine, finasteride, calcium, zopiclone, amoxicillin, clotrimazole, chloramphenicol, citalopram, dexamethasone, pneumococcal vaccine, unspecified formulation, alginic acid, diazepam, lactulose, trimethoprim, clavulanate, folic acid, tiotropium, benzalkonium, lidocaine, potassium, zinc oxide, levothyroxine, lanolin, chlorhexidine, albuterol, oxytetracycline, cetirizine, amitriptyline, sennoside B, mineral oil, tramadol, dihydrocodeine, fusidate, omeprazole, neomycin, clarithromycin, loperamide, phenoxyethanol, ranitidine, naproxen, prochlorperazine, paraffin, cephalexin, nystatin, fluoxetine, diclofenac, erythromycin, isopropyl myristate, loratadine, miconazole, betamethasone, estradiol, clobetasone, timolol, gliclazide, latanoprost, salicylic acid.

The plasmode process was done through modification of an R package on CRAN: Plasmode. The modified code can be found on GitHub.

4 PROPENSITY SCORE MODELLING: CROSS-VALIDATION HYPERPARAMETER TUNING FRAMEWORK

We implemented a 10-fold cross-validation hyperparameter tuning framework. Hyperparameters tuned for each model as below:

XgBoost: number of estimators, minimum sum of instance weight(hessian) needed in a child, minimum loss reduction required to make a further partition on a leaf node of the tree, Subsample ratio of the training instance, the learning rate of boosting, maximum tree depth for base learners.

MLP: optimizer, number of samples per gradient update (batch size), number of epochs to train the model (epochs), kernel function, number of hidden layers, number of units in each layer, activation function.

LASSO: shrinkage parameter

Python codes used to implement these can be found in GitHub.

For the reference model, expert-selected covariates were as follow: drug era observed or not during day -180 through -1 days relative to index for following drugs: diazepam, lactulose, trimethoprim, clavulanate, folic acid, tiotropium, benzalkonium, lidocaine, potassium, zinc oxide, levothyroxine, lanolin, chlorhexidine, albuterol, oxytetracycline, cetirizine, amitriptyline, sennoside B, mineral oil, tramadol, dihydrocodeine, fusidate, omeprazole, neomycin, clarithromycin, loperamide, phenoxyethanol, ranitidine, naproxen, prochlorperazine, paraffin, cephalexin, nystatin, fluoxetine, diclofenac, erythromycin, isopropyl myristate, loratadine, miconazole, betamethasone, estradiol, clobetasone, timolol, gliclazide, latanoprost, salicylic acid, prednisolone, metformin, dihydrocodeine, levothyroxine, tramadol, temazepam, citalopram, diazepam, fluoxetine, codeine, estradiol, dexamethasone, zopiclone, cholecalciferol, condition occurrence observed or not any time prior through -1 days relative to index for the following conditions: Mild chronic obstructive pulmonary disease, Dyspnea, Menopausal flushing, Polymyalgia rheumatica, Fully mobile, Mixed anxiety and depressive disorder, Depressive disorder, Diabetes mellitus, Cerebrovascular accident, Orthostatic hypotension, Type 2 diabetes mellitus, Menopause present, Hypothyroidism, Essential hypertension, Chronic kidney disease stage 3, Hypertensive disorder, Fracture of bone, Fracture of neck of femur, Obesity, Thyrotoxicosis, Cerebrovascular disease, Acute myocardial infarction, Rheumatoid arthritis, Ischemic heart disease, Malignant neoplasm of female breast, Malignant tumor of prostate, Osteoporosis, Chronic kidney disease stage 2, White. Gender, age

For both clinical data and plasmode experiments, we tuned on 50% of the data, following the previous study's considered tuning. Also, to ensure that the training data closely mirrors the original data's treatment prevalence (approximately 0.26 treatment prevalence in the dataset), we implemented a stratification approach when partitioning the data for cross-validation. This means that we organized the data into distinct strata based on the treatment variable for cross-validation, aiming to maintain a consistent distribution of treatments in the training sets.

5 RESULTS

5.1 Real-world Data

The data generation process was summarised in flowchart Figure S3.

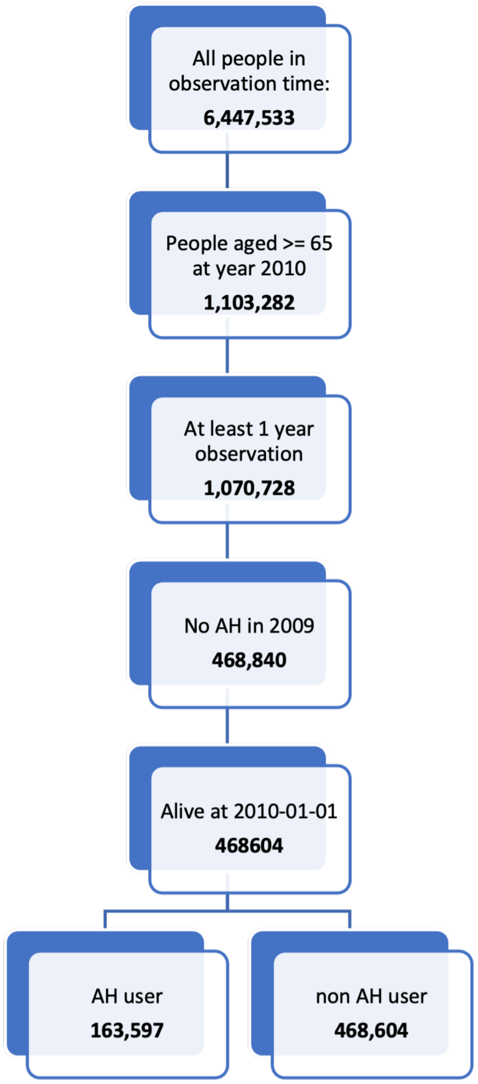

Figure S3. Cohort generation flowchart

5.2 PS and DRS Results

5.3 Brier Score Loss

In the Supplementary Material, we present detailed Brier score loss metrics used during hyperparameter tuning, as discussed in the Method section. For PS estimations, the XgBoost model yielded the most favourable Brier score loss. When evaluating four estimation methods for DRS, the reference method achieved the best Brier score loss in plasmode simulations, whereas XgBoost performed optimally in clinical datasets. Across various data-driven machine learning methods, results were comparable, as indicated by overlapping confidence intervals. Notably, DRS exhibited a lower Brier score loss compared to PS estimations in both clinical and plasmode datasets, a phenomenon attributed to the inherently low outcome risk and the resultant skewed distribution of Brier score loss.

5.4 Covariate Balance

After PS matching in real-world data, there were 4 covariates that had ASMD greater than 0.1 for LASSO estimated PS: drug era during day -180 through -1 days relative to index: ibuprofen, drug era during day

Table S1. Brier score loss in the clinical data analysis and plasmode simulation using different propensity score and disease risk score matching

	Brier score loss			
	DRS clinical dataset	PS clinical dataset	DRS plasmode	PS plasmode
Reference method	0.0074	0.2494	0.0075 (0.0073, 0.0077)	0.1350 (0.1346, 0.1355)
LASSO	0.0074	0.0880	0.0077 (0.0075, 0.0078)	0.1324 (0.1320, 0.1329)
XgBoost	0.0071	0.0556	0.0076 (0.0075, 0.0078)	0.1259 (0.1250, 0.1269)
MLP	0.0075	0.4121	0.0077 (0.0075, 0.0078)	0.1352 (0.1329, 0.1375)

Table S2. Covariate balance for clinical dataset and plasmode simulation: before and after propensity score and disease risk score matching scatterplot of absolute standardized differences

	ASMD DRS plasmode	ASMD PS plasmode	ASMD DRS clinical dataset	ASMD PS clinical dataset
Reference method	0.0973 (0.0961, 0.0986)	0.1032 (0.1020, 0.1045)	0.0426	0.0394
LASSO	0.1223 (0.1192, 0.1253)	0.0991 (0.0978, 0.1003)	0.0442	0.0167
XgBoost	0.1167 (0.1140, 0.1195)	0.0990 (0.0968, 0.1011)	0.0430	0.0150
MLP	0.1007 (0.0988, 0.1026)	0.1010 (0.0992, 0.1027)	0.0703	0.0480

-180 through -1 days relative to index: nitrofurantoin, condition occurrence any time prior through -1 days relative to index: Sinus headache, drug era during day -30 through -1 days relative to index: lansoprazole. After discussion with a clinical epidemiologist, none of these are deemed as a strong confounder, although lansoprazole could be perceived as one, given its association with fracture risk in multiple studies;

There were 29 covariates that had ASMD over 0.1 for matching on MLP estimated PS. Two of them were identified as strong confounders by a clinical epidemiologist (female gender and history of "At risk of osteoporosis"), and one as a potential confounder (drug era during day -30 through -1 days relative to index: lansoprazole);

There were 32 covariates with ASMD over 0.1 for covariates after matching on the reference method estimated PS. Again, some were seen as confounders due to their known association with the outcome of interest (fracture): metformin use, gender, history of Chronic obstructive lung disease, and use of lansoprazole in the previous month.

After DRS matching in real-world data, there were 43 covariates that had ASMD over 0.1 after DRS matching for reference methods. Some of them were identified as confounders after clinical review due to their known association with fracture risk: metformin use, and gender. history of chronic obstructive lung disease, and use of lansoprazole in the previous month.

Similarly, there were 47 covariates that had ASMD over 0.1 for LASSO, with key confounders including use of metformin, use of betamethasone, gender, history of chronic obstructive lung disease, history of "traumatic AND/OR non-traumatic injury", and use of lansoprazole in the previous month.

MLP-estimated DRS led to the highest number of imbalanced covariates: there were 112 that had ASMD greater than 0.1 after matching on MLP-based DRS. Some of these were identified as confounders after clinical review: metformin use, alendronate use, tramadol use, codeine use, gender, history of "At risk of osteoporosis", mild chronic obstructive pulmonary disease, Polymyalgia rheumatica, Alzheimer's disease, chronic obstructive lung disease, traumatic AND/OR non-traumatic injury, history of fracture of neck of femur, C/O - low back pain, and recent use of temazepam, omeprazole, prednisolone, beclomethasone, and lansoprazole in the previous month.

Finally, there were 41 covariates that had ASMD over 0.1 for XgBoost. The most important confounders identified after clinical review included: use of metformin, gender, history of "At risk of osteoporosis", chronic obstructive lung disease, and use of lansoprazole in the previous month.

5.5 Effect Size Results

The results in Table S3 revealed that all hazard ratio estimates for the effect of antihypertensive drugs on fracture risk were greater than 1, indicating a potential positive causal association between antihypertensive drug usage and increased fracture incidence. However, this conclusion was challenged by potential residual

	PS	DRS
Reference	2.34 (2.20, 2.50)	3.92 (3.68, 4.19)
LASSO	2.09 (1.94, 2.56)	3.82 (3.58, 4.08)
XgBoost	1.93 (1.76, 2.11)	3.87 (3.62, 4.13)
MLP	2.28 (2.13, 2.44)	4.24 (3.97, 4.52)

Table S3. Hazard ratio estimations of fracture: before empirical calibration

biases highlighted during the negative control outcome analysis. This analysis raised concerns over the low coverage of negative control outcomes, suggesting that initial hazard ratio estimates could be biased or unreliable due to unaddressed confounding factors.

To address these concerns, empirical calibration was conducted using the OHDSI package https://ohdsi.github.io/EmpiricalCalibration/. This process began by assessing negative control outcomes to quantify the extent of residual bias, which then informed the degree of adjustment required for the initial hazard ratio estimates. The recalibration algorithm statistically adjusted the hazard ratio estimates by applying a bias factor derived from the negative control outcomes comparison, aiming to correct for unmeasured confounding. This recalibration led to hazard ratios that more accurately represent the true causal relationship, with adjustments affecting the HRs, and indirectly, the p-values and standard errors.

	PS	DRS
Reference	2.41 (1.93, 3.03)	4.04 (3.18, 5.13)
LASSO	2.15 (1.73, 2.72)	3.93 (3.14, 4.92)
XgBoost	2.10 (1.68, 2.61)	3.99 (3.19, 4.99)
MLP	2.36 (1.88, 2.95)	4.56 (3.65, 5.69)

Table S4. Hazard ratio estimations of fracture: after empirical calibration

Following recalibration, results are shown in Table S4, all hazard ratio estimates decreased, with both PS-estimated and DRS-estimated hazard ratios remaining above 1. This adjustment suggests a more nuanced interpretation of the causal relationship between antihypertensive drug use and fracture risk, highlighting

the importance of addressing potential biases in observational data. However, the conclusion of a positive causal correlation between antihypertensive drug and fracture remains the same after calibration.
